# Resolving the relationships of clams and cockles: dense transcriptome sampling drastically improves the bivalve tree of life

**DOI:** 10.1098/rspb.2018.2684

**Published:** 2019-02-06

**Authors:** Sarah Lemer, Rüdiger Bieler, Gonzalo Giribet

**Affiliations:** 1 University of Guam Marine Laboratory, 303 University Drive, UOG Station, Mangilao, GU 96923, USA; 2 Museum of Comparative Zoology, Department of Organismic and Evolutionary Biology, Harvard University, 26 Oxford Street, Cambridge, MA 02138, USA; 3 Integrative Research Center, Field Museum of Natural History, 1400 South Lake Shore Drive, Chicago, IL 60605, USA

**Keywords:** Imparidentia, phylogenomics, phylogenetics, Mollusca, Bivalvia

## Abstract

Bivalvia has been the subject of extensive recent phylogenetic work to attempt resolving either the backbone of the bivalve tree using transcriptomic data, or the tips using morpho-anatomical data and up to five genetic markers. Yet the first approach lacked decisive taxon sampling and the second failed to resolve many interfamilial relationships, especially within the diverse clade Imparidentia. Here we combine dense taxon sampling with 108 deep-sequenced Illumina-based transcriptomes to provide resolution in nodes that required additional study. We designed specific data matrices to address the poorly resolved relationships within Imparidentia. Our results support the overall backbone of the bivalve tree, the monophyly of Bivalvia and all its main nodes, although the monophyly of Protobranchia remains less clear. Likewise, the inter-relationships of the six main bivalve clades were fully supported. Within Imparidentia, resolution increases when analysing Imparidentia-specific matrices. Lucinidae, Thyasiridae and Gastrochaenida represent three early branches. Gastrochaenida is sister group to all remaining imparidentians, which divide into six orders. Neoheterodontei is always fully supported, and consists of Sphaeriida, Myida and Venerida, with the latter now also containing Mactroidea, Ungulinoidea and Chamidae, a family particularly difficult to place in earlier work. Overall, our study, by using densely sampled transcriptomes, provides the best-resolved bivalve phylogeny to date.

## 1. Introduction

Bivalvia is among the most diverse molluscan classes, totalling almost 10 000 described extant species (see [1]) inhabiting various aquatic environments, spanning freshwater, brackish and marine, as well as ranging from the shallow continental shelve to the deep sea, including hydrothermal vents and hydrocarbon seeps. Bivalves thus adopt a multitude of different life modes, from detritivory to filter feeding, with extreme cases of photo- and chemosymbiosis to carnivory [2–5]. Many species of bivalves constitute an important food source and have a role in culture and folklore [6], or even in the medical and bioengineering fields [7–9]. Bivalves are also being increasingly used to study the spread of cancer in natural environments, as a model of contagious tumours [10], and to assess gene expression of cancer-related genes [11]. Because of their filter feeding habits, they also hold a major function in coastal ecosystems and reef ecology [12,13]. As a result their ecology, taxonomy, population genetics and phylogenetic relationship have been intensely studied at the morphological and molecular level (see [14] for a recent review), including transcriptomic approaches [15,16].

While the phylogenetic backbone of bivalves is relatively well resolved [15], numerous uncertainties, especially within its large clade, recently named Imparidentia (equivalent to Euheterodonta excluding Anomalodesmata; Bieler *et al*. [17]), remain, probably due to lack of information in Sanger-based approaches (e.g. [14,17–19]), or due to the lack of taxon sampling in phylogenomic approaches [15]. Many subgroups of bivalves have been relatively well defined by morpho-anatomical characters (e.g. unionid freshwater mussels, mytilid mussels, venerid clams), but the relationships of these and many other putative larger clades within the bivalve tree were long contentious. Numerous bivalve tree patterns were proposed, based on the chosen morphological character system or available molecular markers of a particular study (see discussions in [19,20]). A total-evidence analysis based on a large morphological dataset in combination with up to nine molecular markers [17] proposed a new topology of the bivalve tree, with six major clades (Protobranchia, Pteriomorphia, Archiheterodonta, Palaeoheterodonta, Anomalodesmata, Imparidentia) that have been adopted at the levels of subclasses and superorders in current ranked bivalve classifications [21]. The 2014 study, however, could not find full support for all deeper nodes of the tree. This included the question of monophyly of the Protobranchia (discussed in [22], based on larger taxon sampling), and of the monophyletic versus paraphyletic branching pattern of Archiheterodonta + Palaeoheterodonta (subsequently addressed by [15]).

Imparidentia, one of the major six clades of living Bivalvia introduced by Bieler *et al*. [17], encompasses the majority of mostly marine bivalve families and spans many well-known and economically important groups such as cockles, venus clams, giant clams and shipworms. The structure of this large clade has, however, remained unresolved. It clearly contains the order Lucinida (with or without the family Thyasiridae) and the large Neoheterodontei clade (first defined by [23]) that includes the freshwater Sphaeriidae and the two major orders Myida and Venerida. However, a large number of imparidentian families could not be placed with certainty and some, such as the extremely long-branched Chamidae (jewel box clams), proved particularly vexing in the analyses. A subsequent study based on a 5-gene Sanger-based approach [14] provided much expanded taxon sampling for the imparidentian families, but—as in all prior Sanger-based studies (e.g. [19,23])—again could not resolve the positions of families such as Chamidae and Gastrochaenidae. The latter family was, however, included in the phylogenomic analysis of González *et al*. [15], where it found support as sister group of the non-lucinid imparidentians. That study demonstrated the utility of phylogenomic approaches to resolving such nodes in bivalve phylogeny but had limited taxon sampling and did not include other problematic taxa such as Chamidae and Thyasiridae.

To resolve the internal structure of this major branch of Bivalvia that remained opaque to morpho-anatomical and Sanger-based approaches, we analyse 99 bivalve transcriptomes (59 newly sequenced in this study) together with nine molluscan outgroups to explore the remaining uncertainties of Bivalvia's phylogenetic relationships both for deep and shallow divergent nodes. In order to do so, we apply specific orthology searches to optimize the generation of data matrices for different evolutionary questions (whole Bivalvia versus Imparidentia matrices), and implement analytical methods well known to ameliorate common biases in phylogenomic analyses.

## 2. Material and methods

### (a) Taxon sampling, cDNA library construction and next-generation sequencing

A total of 108 samples were analysed in this study: 99 bivalves (98 species) and nine non-bivalve mollusc outgroups. We sequenced cDNA from 59 specimens using an Illumina HiSeq 2500 platform, and combined these with 44 transcriptomes previously sequenced in our laboratory [15,16], and five publicly available transcriptomes including one genome (see electronic supplementary material, table S1 and the MCZ online collections database, http://mczbase.mcz.harvard.edu). When compared to the phylogenomic analysis of González *et al*. [15], we have tripled the number of included imparidentian species from 17 to 52.

All tissues were collected fresh and immediately flash frozen in liquid nitrogen or fixed in RNA*later* (Life Technologies, Carlsbad, CA, USA) and stored at −80°C. Total RNA was extracted using TRIzol (Life Sciences) and purification of mRNA was performed using the Dynabeads (Invitrogen) following the manufacturer's instructions and as described in Lemer *et al*. [16]. For each sample, mRNA was eluted in 15 ml of Tris-HCl buffer, quality assessed with a picoRNA assay in an Agilent 2100 Bioanalyzer (Agilent Technologies) and quantity measured with an RNA assay in a Qubit fluorometer (Life Technologies).

All cDNA libraries were constructed using the PrepX mRNA kit for Apollo 324 (Wafergen) by inputting approximately 100 ng of RNA per sample in the instrument. Each library was barcoded with TruSeq single indices (i7) to allow multiplexed sequencing runs. Each library concentration was measured by a real time qPCR run on a MX3000P qPCR system (Agilent Technologies) using the Kapa Library quantification kit for NGS (Kapa Biosystems); quality and size selection were assessed with an HS DNA assay in an Agilent 2100 Bioanalyzer (Agilent Technologies) (final library concentration varied between 5 nM and 200 nM). Libraries were then sequenced on the Illumina HiSeq 2500 platform with paired-end reads of 150 bp at the FAS Center for Systems Biology at Harvard University.

### (b) Transcriptome assembly

All reads generated for this study are deposited in the National Center for Biotechnology Information Sequence Read Archive (NCBI-SRA; electronic supplementary material, table S1). Demultiplexed Illumina HiSeq 2500 sequencing results were retrieved in FASTQ format from the sequencing facility (Bauer Core—Harvard University) and in SRA format from GenBank. Each sample, except for the genome of *Lottia gigantea*, was prepared as in Lemer *et al*. [16]. In brief, reads were filtered for quality, adapters and rRNA contamination using Trimgalore version 0.3.3 [24] and Bowtie 2.0.0 [25]. The protein assembly of the *Lottia gigantea* genome was downloaded from the EMBL database (http://metazoa.ensembl.org/Lottia_gigantea/Info/Index).

*De novo* transcriptome assemblies were conducted for each sample with Trinity r2014-04-13 [26,27] using paired read files and default parameters except for --path_reinforcement_distance 50. Reduction of redundant transcripts was done in each transcriptome and genome with CD-HIT version 4.6 [28] using a threshold of 98% global similarity. Predicted peptides for each transcriptome were identified with TransDecoder 3.0.0 [27] with default settings and filtered for isoforms with a custom Python script.

### (c) Orthology assignment and matrix construction

Orthology assignment across all assembled transcriptomes was performed using stand-alone OMA 1.06 [29,30]. The parameters.drw file retained all default settings with the exception of ‘MaxTimePerLevel’, which was set at 3600, to optimize the software for our server cluster. The all-by-all local alignment process was parallelized across 400 CPUs once all the input pre-processing steps were achieved on a single core (to avoid risk of collision). All 234 663 orthogroups were aligned individually using MUSCLE 3.6 [31]. Divergently aligned positions were culled by a probabilistic character masking approach with ZORRO [32], using default parameters and FastTree 2.1.4 [33] to construct guide trees. In all of the alignments, positions that were assigned a confidence score below the threshold of 5 by ZORRO were discarded, using a custom Python script.

Two initial data matrices following occupancy thresholds [34] were generated for phylogenetic analyses, using a custom Python script: the first one, *Matrix 1*, targeting a minimum gene occupancy of 50%, was constructed by selecting the OMA orthogroups present in 55 or more taxa (resulting in 312 orthogroups). The second matrix, *Matrix 2* includes orthogroups present in 77 or more taxa (gene occupancy greater than 70%; resulting in 102 orthogroups). Selected orthogroups for each matrix were then concatenated using Phyutility 2.6 [35].

To explore the complex topology of the Imparidentia subclade, a second orthology assignment run was performed using OMA 2.0, with taxa from the Imparidentia clade only (52 taxa) and six outgroups (see electronic supplementary material, table S1 for details). The objective of this approach was to design matrices optimized for Imparidentia rather than subsamples of the original dataset (as done in [36]). The 137 741 orthogroups obtained were filtered and prepared as described above. *Matrix 3i* was constructed by retaining orthogroups with minimum gene occupancy of 50% (28 or more taxa; resulting in 439 orthogroups).

To account for potential biases based on gene evolutionary rates on the Imparidentia tree topology, *Matrix 4i* was constructed by discarding the orthogroups with the 20% highest and 20% lowest evolutionary rates of *Matrix 3i*. Orthogroups were sorted based on their evolutionary rate using per cent pairwise identity as a proxy. Accumulated conservation values were generated for each locus using Trimal 1.2b (-sct flag). Loci were sorted; the first being the slowest evolving genes (most conserved) and the last being the fastest evolving genes (least conserved). A total of 343 orthogroups were retained, for a matrix with 37% missing data.

### (d) Phylogenetic analyses

Maximum-likelihood inference was computed for *Matrices 1*, *3i* and *4i* with RAxML 7.7.5 [37] using PROTGAMMALG4X as the best-fit model of amino acid substitution and 100 bootstrap replicates on concatenated orthogroups. Additionally, maximum-likelihood inference was also computed for *Matrices 1*, *3i* and *4i* with partitioned data using IQtree 1.6.1 [38–40]. We included the ModelFinder option [41] which automatically selects the best-fit model for each partition (i.e. orthogroup) and the -sq flag which allows each partition to have its own set of branch lengths, thus accounts for heterotachy [42]. Three independent runs were conducted for each matrix, each with 1000 ultrafast bootstrap replications, which resamples site within partitions, and the most likely tree was retained [43]. Maximum-likelihood inferences for *Matrix 2* were computed using a principal component approach to improve amino acid substitution matrices (PCMA) with PhyML 3.0 [44]; this computational intensive method could only be used for this smaller matrix. Analyses were conducted with 10 principal components and three random starting trees for each run.

*Matrices 2* and *3i* were also analysed using Bayesian inference with PhyloBayes MPI 1.7a with openmpi 1.10 [45] using the site-heterogeneous CAT-GTR model of evolution [46]. Three independent Markov chain Monte Carlo (MCMC) runs were conducted for 7688–11 551 cycles (*Matrix 2*) and 7152–12 252 cycles (*Matrix 3i*). The initial cycles in each MCMC run were discarded as burn-in and determined using the ‘tracecomp’ executable. Convergence was assessed using the ‘bpcomp’ executable, and chains were considered to have converged when the maximum bipartition discrepancies (maxdiff) across a minimum of two independent chains reached 0.2.

To test for putative gene incongruence within Imparidentia we inferred individual gene trees for each orthogroup included in *Matrix 3i* using RAxML 7.7.5. PROTGAMMALG4X was selected as the best-fit model of amino acid substitution. All individual best-scoring trees were concatenated for each matrix and fed into SuperQ 1.1 [47] in order to visualize inter-gene conflicts. SuperQ decomposes all gene trees into quartets to infer a super-network where edge lengths are assigned based on quartet frequencies; it was run using the ‘balanced’ edge-weight optimization function with no filter. The resulting super-networks were visualized with SplitsTree 4.13.1 [48].

Finally, to minimize the potential impact of compositional heterogeneity and long-branch attraction (e.g. [49]), we recoded *Matrices 1* and *3i* into Dayhoff categories [50]; thus reducing the 20 character states of amino acids down to six states [51]. We assigned the following numbers to each amino acid: 0: AGPST, 1: FWY, 2: C, 3: HKR, 4: ILMV, 5: EDNQ. The recoded matrices were analysed with RAxML 7.7.5 using a multi-state model (-m MULTIGAMMA -K GTR).

## 3. Results and discussion

### (a) Strengthening the phylogenetic backbone of Bivalvia

Although the backbone of the bivalve tree of life has been explored extensively in recent times (e.g. [14,17,19,52–57]), a number of uncertainties remain. The phylogenetic dataset generated in this study is the largest ever gathered to attempt to resolve these relationships. We analysed 108 transcriptomes and genomes and explored four matrices using both maximum-likelihood and Bayesian approaches. The orthology assessment of the 108-taxon dataset with the OMA stand-alone algorithm generated 234 663 orthogroups. Details of the values used to assess the quality of the assembled transcriptomes (number of sequenced reads, used reads and contigs) as well as accession numbers, can be found in the electronic supplementary material, table S1. Concatenated matrices were compiled using a threshold of per cent gene occupancy. Both *Matrix 1* and *Matrix 2* contained data for all the taxa included in the study, though each taxon varied in gene representation (electronic supplementary material, figure S1). The two main matrices constructed yielded 312 (*Matrix 1*: occupancy of more than 50%, 70 488 aa) and 102 (*Matrix 2*: occupancy of more than 70%, 22 164 aa) orthologues, respectively.

**Figure 1.**
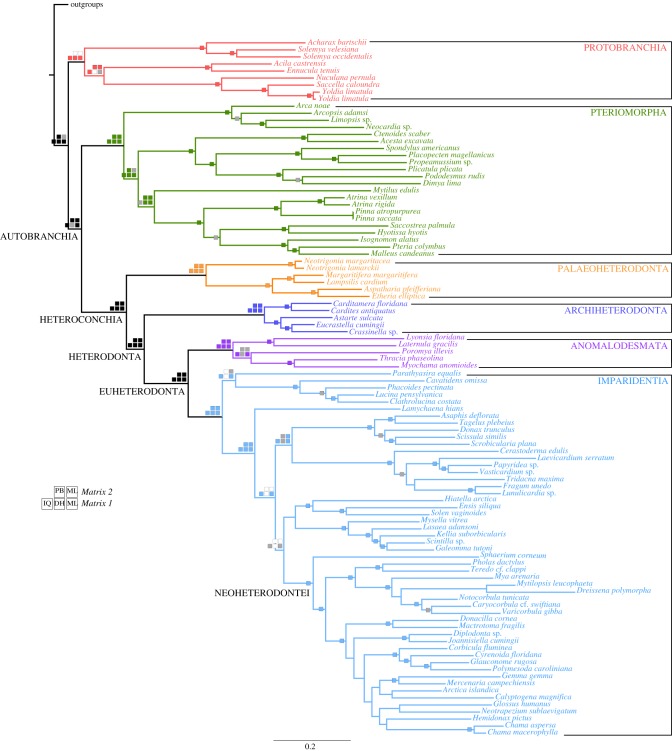
Phylogenetic hypothesis for Bivalvia based on *Matrix 1* analysed in RAxML with support values plotted as follows: checked boards in major deep nodes represent nodal support for the different analyses in *Matrix 1* (bottom row) and *Matrix 2* (top row). PHYML-PCMA and RAxML are abbreviated as ML, PHYLOBAYES: PB, RAxML with Dayhoff recoding: DH and IQtree: IQ. Filled squares indicate nodal support values higher than 90% bootstraps (ML) and a posterior probability of 0.99 or higher (PB). Grey squares indicate lower nodal support and white squares indicate unrecovered nodes in the specified analysis. Single squares on internal nodes indicate that the node was recovered in all five analyses either with maximum or partial support. Internal nodes not recovered by all five analyses are not reported. Bivalvia subclasses are represented in different shades of colour.

All the phylogenetic analyses conducted on the two main matrices revealed a well-supported topology for all deep nodes in the bivalve tree of life (figure 1). Every analysis conducted with the two main matrices recovered monophyly of Pteriomorphia, Heteroconchia, Palaeoheterodonta, Euheterodonta, Archiheterodonta, Anomalodesmata, Imparidentia and Neoheterodontei with full support (100% bootstrap or posterior probability of 1; figure 1). The relationships among the heteroconchian clades were also consistent across all analyses, with Palaeoheterodonta as sister group to Heterodonta, Archiheterodonta as sister group to Euheterodonta, and Anomalodesmata as sister group of Imparidentia, as in most recent studies [14,15], but contradicting earlier findings of a clade including Palaeoheterodonta and Archiheterodonta (e.g. [17,19]) and results based on mitogenomic data (e.g. [56,57]).

Monophyly of Protobranchia was recovered in all analyses conducted with *Matrix 1* (figure 1). However, in analyses conducted with *Matrix 2*, Solemyoidea appears as sister group to all other bivalves, rendering Protobranchia paraphyletic. The latter result contrasts with most recent bivalve phylogenetic studies [15,19], which recovered monophyly of Protobranchia in all their analyses, although in the phylogenomic analyses of González *et al*. [15], some gene conflict was detected. Here we expanded the sampling within Protobranchia from three to nine transcriptomes (belonging to eight species), which continued to support monophyly only in the analyses of *Matrix 1*. The increased taxon sampling, however, allowed us to clarify the relationships among the protobranch superfamilies, Nuculoidea being sister group of Nuculanoidea in most analyses (figure 1), as recognized in earlier work [19,22]. Non-monophyly of Protobranchia was also reported in the bivalve phylogenetic study of Combosch *et al*. [14], among many other molecular studies, where the authors included 25 protobranch taxa and recovered Solemyoidea clustering with the outgroups. The reasons for the non-recovery of the subclass Protobranchia under some analytical conditions remain puzzling and highlight the necessity for a deeper phylogenomic focus on this group, especially including members of the unsampled Manzanelloidea (Manzanellidae and Nucinellidae), but could also be attributed to the relatively small number of genes (102) of *Matrix 2*.

Relationships within Pteriomorphia are entirely consistent with the most recent phylogenomic analyses focusing on this clade [16] and were well supported in all our analyses but one (figure 1). Mytilida was placed as a sister group to Ostreida (Pinnidae, Ostreoidea and Pterioidea) in all analyses (although with low nodal support in the IQtree analyses). Arcida appeared as sister group to all other pteriomorphians in all analyses with maximum nodal support except in the PhyML-PCMA tree (with 80% bootstrap support). For further details on the history and hypotheses of pteriomorphians see Lemer *et al*. [16].

For Heteroconchia, our phylogenomic analyses recovered a similar topology to that of González *et al*. [15]; i.e. a first split between Palaeoheterodonta and Heterodonta; Archiheterodonta as sister group of Euheterodonta; and a main division of Euheterodonta into Anomalodesmata and Imparidentia. The well-supported Palaeoheterodonta segregated in all analyses, as expected, in two main clades; the marine Trigoniida and the freshwater Unionida. Within Unionida, Unionidae and Margaritiferidae always clustered together (i.e. Unionoidea) and likewise, Iridinidae and Etheriidae (i.e. Etherioidea) always formed a clade (figure 1). The current sampling does not permit addressing detailed phylogenetic and biogeographic questions within Unionida.

Archiheterodonta, a clade composed of what some authors have considered the most ‘primitive’ heterodonts, based on morphological characters such as sperm [58], periostracum formation, and extracellular high molecular weight haemoglobin [59,60], was recovered as sister group to all other Heterodonta (i.e. Euheterodonta) in all our analyses, as previously found by González *et al*. [15]. Our sampling enabled us to support two superfamilies, Carditoidea (Carditidae) and Crassatelloidea (Crassatellidae + Astartidae), as in the most recent analysis of Archiheterodonta [53] (figure 1).

Anomalodesmata was the sister clade to Imparidentia in all analyses with high support values (figure 1). Within this clade we constantly recovered a deep subdivision of analysed taxa into two groups mostly corresponding to the ‘lyonsiid’ and ‘thraciid’ lineages of Harper *et al*. [61], as also seen in subsequent phylogenetic analyses including sufficient taxon sampling of anomalodesmatans [14,17,23], as well as in studies of sperm ultrastructure [62]. The ‘lyonsiid’ samples include members of the families Laternulidae and Lyonsiidae and the ‘thraciid’ lineage members of Thraciidae and Myochamidae. Our sole representative of the carnivorous septibranch families, *Poromya illevis* (Poromyidae), was recovered as the sister group to the ‘thraciids’ in all our analyses except for the Bayesian analyses with *Matrix 2* and the Dayhoff analyses with *Matrix 1*, where it placed as sister group to the ‘lyonsiids’, albeit without significant nodal support in both instances. Monophyly of the three septibranch families (Poromyidae, Cuspidariidae and Verticordiidae) has been rejected in most recent phylogenetic analyses [14,61] and deserves to be explored further, something we could not attempt here due to lack of suitable tissue for transcriptomes.

### (b) Resolving the Imparidentia puzzle

**Figure 2.**
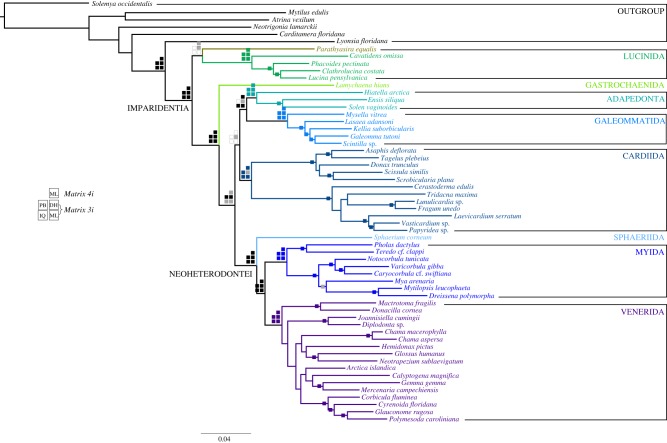
Phylogenetic hypothesis for Imparidentia based on *Matrix 3i* and *4i* analysed in RAxML with and without Dayhoff recoding respectively. Support values plotted as follows: checked boards in major deep nodes represent nodal support for the different analyses in *Matrix 3i* (two bottom rows) and *Matrix 4i* (top row). RAxML is abbreviated as ML, PhyloBayes: PB, RAxML with Dayhoff recoding: DH and IQtree: IQ. Filled squares indicate nodal support values higher than 90% bootstraps (ML) and a posterior probability of 0.99 or higher (PB). Grey squares indicate lower nodal support and white squares indicate unrecovered nodes in the specified analysis. Single squares on internal nodes indicate that the node was recovered in all five analyses either with maximum or partial support. Internal nodes not recovered by all five analyses are not reported. Imparidentia orders are represented by different shades of colour.

**Figure 3.**
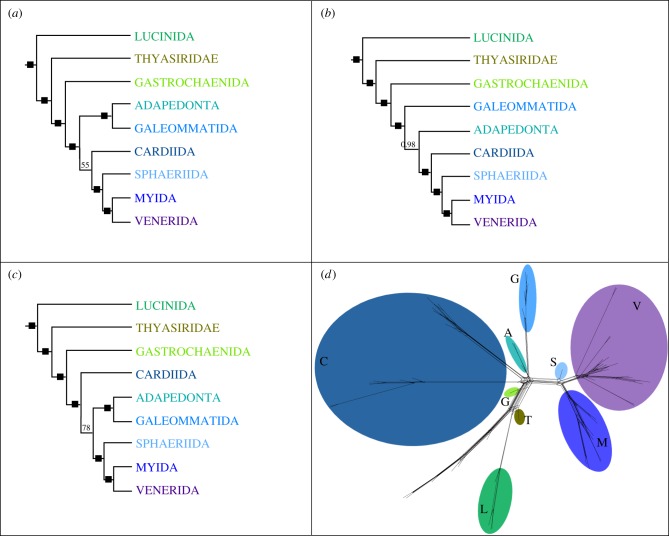
Alternative tree topologies of Imparidentia recovered with *Matrix 3i*: (*a*) RAxML, (*b*) PhyloBayes, (*c*) IQtree, (*d*) Supernetwork. Filled squares indicate nodal support values higher than 90% bootstraps and a posterior probability of 0.99 or higher. Taxa in supernetwork are represented by the first letters of taxa name and the same colour code as in trees *a*, *b* and *c*.

Imparidentia is supported as a clade in nearly all recent phylogenetic analyses of bivalves, yet its internal relationships have remained largely obscured by a combination of factors, including lack of genetic information, deficient taxon sampling, or both, and portrayed as an example of a rapid imparidentian radiation. However, recent analyses have shown a steady diversification through the Palaeozoic and Mesozoic [17]. Nevertheless, and to avoid possible compressions of the deepest branches in the clade, we built a data matrix optimized for Imparidentia. The two matrices enriched for Imparidentia yielded 439 (*Matrix 3i*; figures 2 and 3) and 343 (*Matrix 4i*) orthologues, respectively.

Eight superfamilies of extant Imparidentia were previously placed outside the ordinal framework of Adapedonta, Cardiida, Lucinida, Myida and Venerida [17]. Of these, the superfamily Gastrochaenoidea (with the single family Gastrochaenidae) is the sister group to all other non-lucinid imparidentians and is here elevated to order Gastrochaenida (a concept already used by [63], who had used a suborder ‘Gastrochaenoidea’ for this family). Galeommatoidea (a group including many nominal family-group taxa including Basterotiidae, Galeommatidae [here studied], and Lasaeidae) is here interpreted as order Galeommatida. Sphaerioidea (with family Sphaeriidae) has long been recognized as the most basal member of Neoheterodontei and is here elevated to the ordinal level as Sphaeriida. Mactroidea (with families Mactridae and Mesodesmatidae; Anatinellidae and Cardiliidae not sampled), Ungulinoidea (with family Ungulinidae), and the long-debated Chamoidea (with family Chamidae) are recognized as members of Venerida. Not currently placed, because suitable material was unavailable to this study, are the members of nominal superfamilies Cyamioidea (Cyamiidae, Galatheavalvidae, Sportellidae) and Gaimardioidea (Gaimardiidae).

The first offshoots of Imparidentia comprise Lucinidae and Thyasiridae, but whether these form a clade (i.e. as monophyletic Lucinida; figures 1 and 2) or a grade, with Lucinidae as sister group to Thyasiridae plus the remaining Imparidentia (figure 3), remains elusive. The next branch of the Imparidentia tree is represented by Gastrochaenida as the sister group to all the remaining imparidentians. The latter in some analyses divides into two main groups, a well-supported clade Neoheterodontei including Sphaeriida, Myida and an array of families that we assign to a redefined Venerida, and a second putative clade including Adapedonta, Galeommatida, and a well-supported Tellinoidea–Cardioidea clade (= Cardiida) (figure 2). However, the relationships of Adapedonta, Galeommatida and Cardiida remain unstable (figures 2 and 3), although most analyses, except for PhyloBayes, support a sister group relationship of Adapedonta and Galeommatida. In the maximum-likelihood and Bayesian analyses, Cardiida is sister group to Neoheterodontei with 55% BS and 1.0 pp respectively; whereas in the IQtree analysis Cardiida is placed as sister group to Adapedonta, Galeommatida, and Neoheterodontei with maximum support. When recoding the amino acid matrix with Dayhoff categories, Cardiida is recovered as sister group to the clade formed by Adapedonta and Galeommatida, albeit without significant nodal support. In the Bayesian analysis Galeommatida is sister group to the clade composed of Adapedonta, Cardiida and Neoheterodontei.

Given the current resolution and the composition of the imparidentian clades, we recognize eight orders for extant taxa: Lucinida (with or without the Thyasiridae, which may end up constituting a ninth order), Gastrochaenida, Adapedonta, Galeommatida, Cardiida, Sphaeriida, Myida and Venerida. The higher-level structure of Neoheterodontei appears well resolved, with Myida and Venerida well supported as sister taxa and Sphaeriida being their most immediate outgroup. Resolution within Myida is likewise stable and highly supported; however, Venerida shows more uncertainty, even after the addition of Mactroidea, Ungulinoidea and Chamoidea. The position of Chamidae, a long-standing question in bivalve phylogeny (e.g. [14,17]), is well resolved as a member of Venerida, as it appears within this clade in all analyses and with full support. Its definitive position is less clear. In most analyses, including those for the Imparidentia dataset and using methods that take into account heterotachy and heterogeneity, Chamidae groups with Hemidonacidae, Glossidae, and Trapezidae (e.g. figures 1 and 2). A suite of families within the Neoheterodontei (Glossidae, Hemidonacidae, Glauconomidae, Trapezidae, Arcticidae, Vesicomyidae, Kelliellidae) has been problematic to resolve in prior morphological and Sanger-approach studies (e.g. [14,23,64,65]). Of these, Glauconomidae was found to form a well-supported clade with Cyrenidae and Cyrenoididae and these currently are considered to constitute the superfamily Cyrenoidea [17,19,66], a position here supported. The position of Hemidonacidae remains unresolved and its status as a separate superfamily (e.g. [65]) is here maintained. Formal bivalve classifications [67,68] grouped the remaining families in two superfamilies, Arcticoidea (with Arcticidae and Trapezidae) and Glossoidea (with Glossidae, Kelliellidae and Vesicomyidae). Transcriptomic data do not support these putative clades, with Trapezidae here grouping with Glossidae, and Vesicomyidae (*Calyptogena magnifica*) clustering not with Glossidae but with Arcticidae and Veneridae. An intriguing result is the separation of the putative members of Cyrenidae in the transcriptomic studies (also González *et al*. [15]; *Glauconome* not studied therein). Cyrenidae is generally considered [69] a monophyletic group spanning smaller-shelled freshwater taxa such as *Corbicula* and larger-shelled and mostly estuarine and near-shore mangrove taxa such as *Polymesoda* and *Geloina*. However, results of the current study place *Corbicula* stemward in the Cyrenoidea and *Polymesoda* in its crown group and sister group to *Glauconome*. Investigation of other nominal cyrenids (*Batissa*, *Cyanocyclas*, *Geloina*, *Villorita*) will be needed to fully resolve this group.

Recent work on bivalve phylogeny called for the need of resolving the phylogenetic position of ‘strange’ taxa, such as Chamidae, Gastrochaenidae or Thyasiridae [14,15]. Our new approach strongly supports Gastrochaenidae as the sister group to all other Imparidentia except Lucinida and Thyasiridae. The position of Thyasiridae is reduced to two possibilities, being the sister group (or member) of Lucinida or the sister group to Gastrochaenida plus the remaining imparidentians. Chamidae is less precisely positioned, but it is clearly a member of the redefined Venerida, probably related to Hemidonacidae, Glossidae and Trapezidae. Work continues to be needed to refine such last branches of the bivalve tree of life (including further testing the monophyly of Protobranchia), however we are now far from the time when bivalve families jumped 10 or more nodes up or down the backbone of the tree with each new analysis. New refinements may require increasingly lower-level adjustments of hypothesized branching patterns but we are a lot closer to a stable reconstruction of the bivalve tree of life, a task that seemed daunting only a decade ago.

## Supplementary Material

Table S1: list of analyzed species

## Supplementary Material

Supplementary figure S1: Matrice gene occupancy

## Data accessibility

All reads generated for this study are deposited online in the NCBI-SRA repository and all transcriptome accession and museum catalogue numbers are listed in the electronic supplementary material, table S1. Detailed information about the sampled specimens can be found on the MCZ online collections database: http://mczbase.mcz.harvard.edu. All in-house scripts, assembled transcriptomes, TransDecoder translations and final data matrices can be found in https://dataverse.harvard.edu/dataverse/Bivalve_phylogenomics.
