# Supplementary material for: Resolving the relationships of clams and cockles: dense transcriptome sampling drastically improves the bivalve tree of life
Source: Proc Biol Sci. 2019 Feb 6;286(1896):20182684. doi: 10.1098/rspb.2018.2684 (PMC6408618; doi:10.1098/rspb.2018.2684)

Supplementary figure 1.
Gene occupancy representation per species for each matrix built with an in-house script, available for download. A white cell indicates a gene that was not sampled. Taxa are sorted from the highest (top) to lowest (bottom) gene representation. Species name are coded as in supplementary material table S1.

a. *Matrix 1*

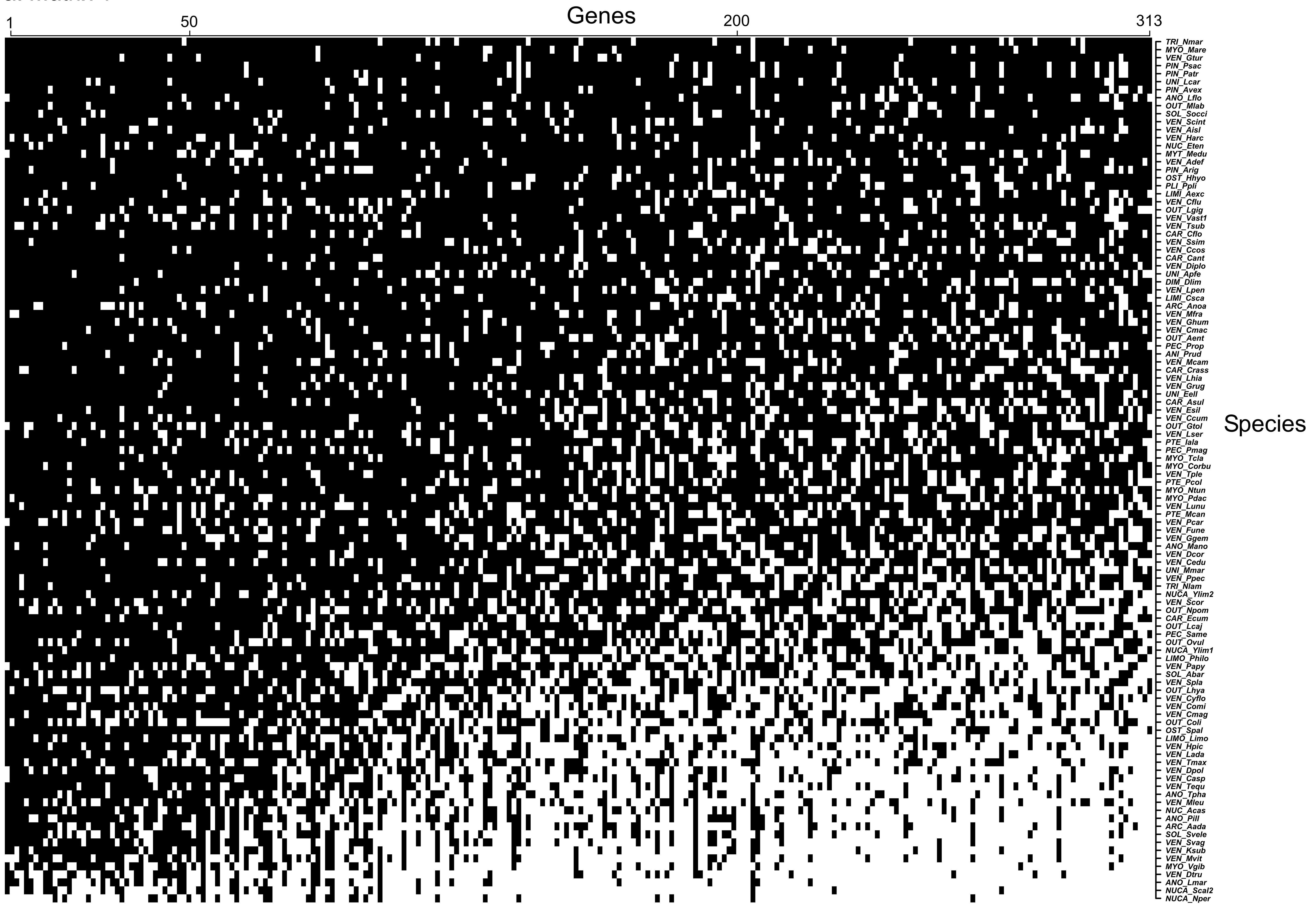

b. *Matrix 2*

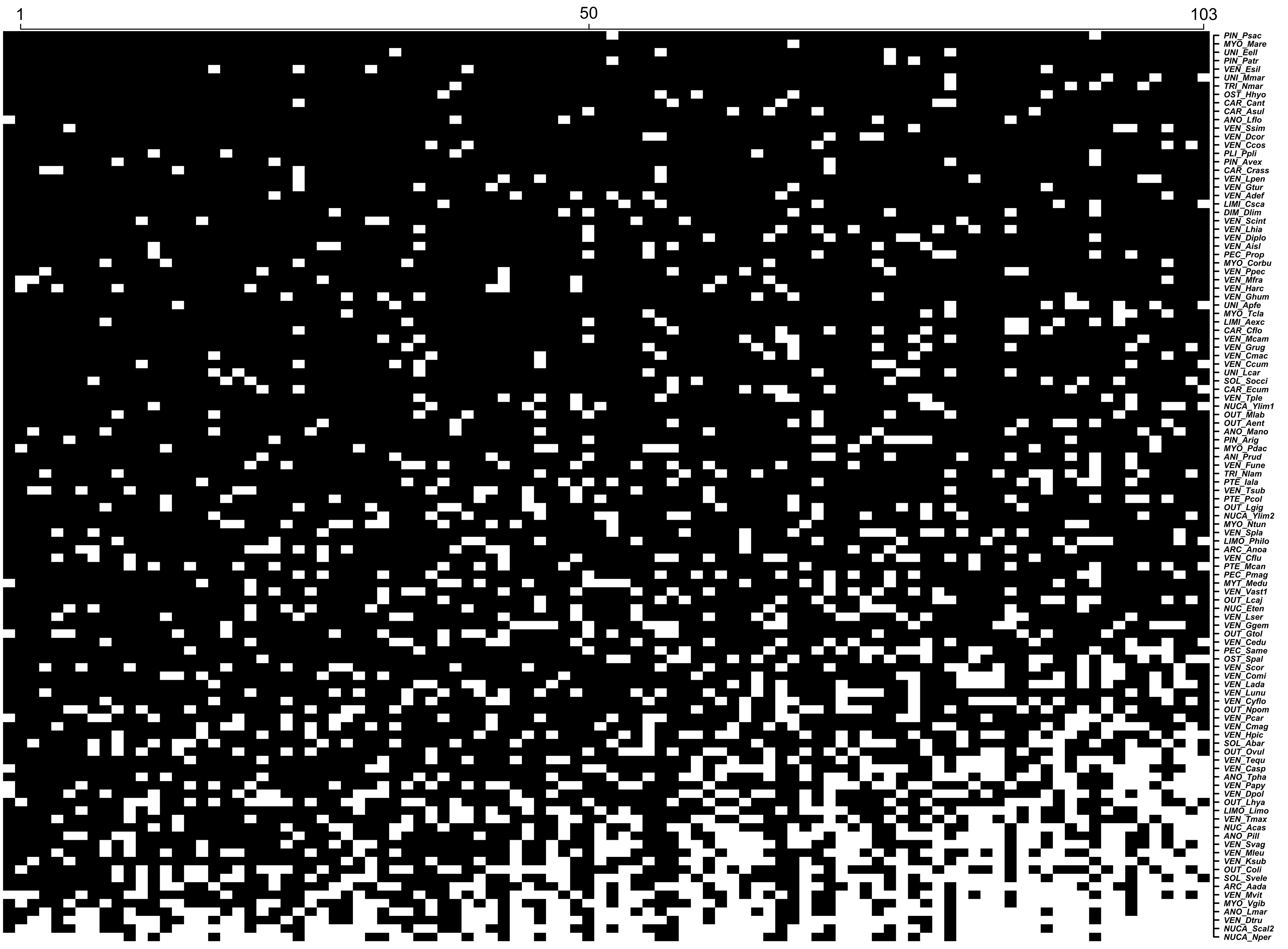

c. *Matrix 3i*

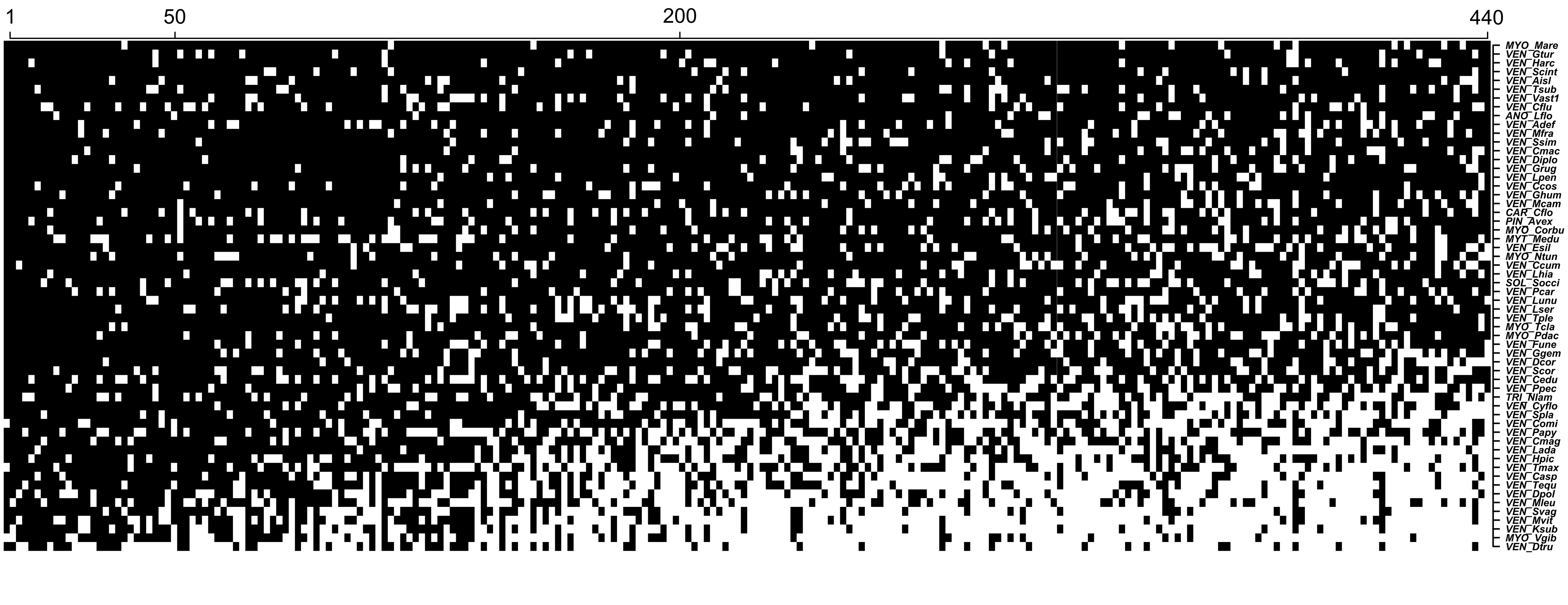

d. *Matrix 4i*

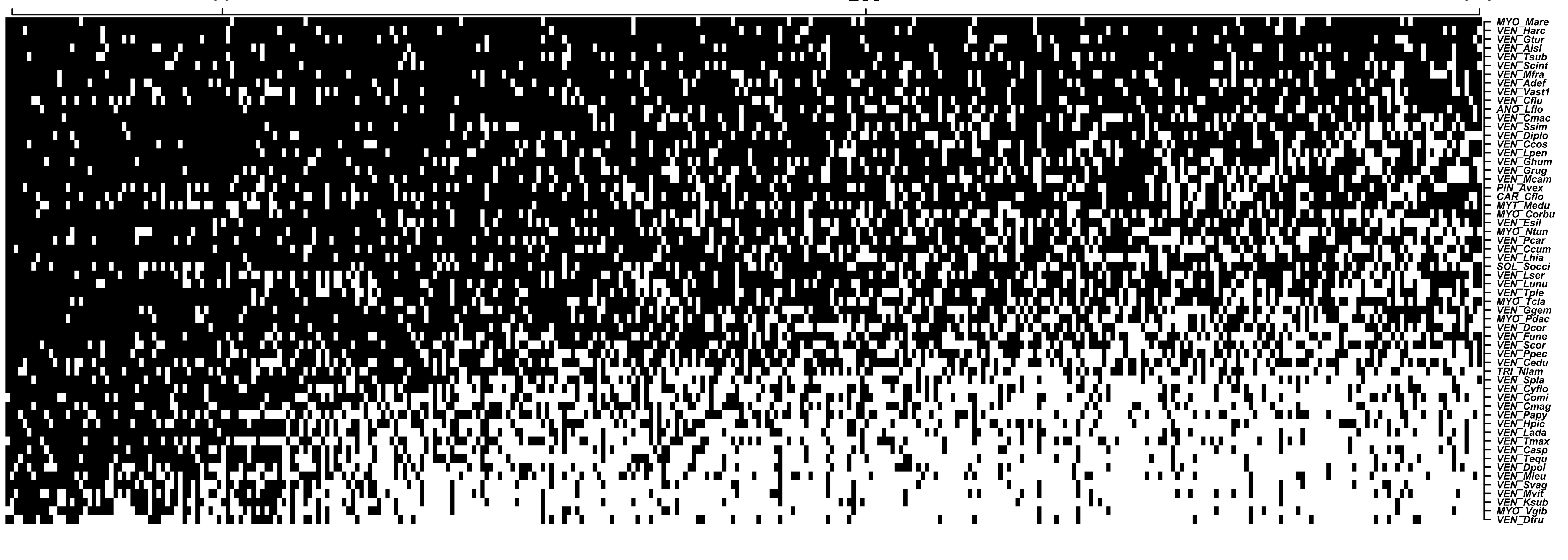

Supplement: Supplementary figure S1: Matrice gene occupancy [file rspb20182684supp2.pdf]
